# Important risk factors and attributable risk of vertebral fractures in the population-based Tromsø study

**DOI:** 10.1186/1471-2474-13-163

**Published:** 2012-08-31

**Authors:** Svanhild Waterloo, Tuan Nguyen, Luai A Ahmed, Jacqueline R Center, Bente Morseth, Nguyen D Nguyen, John A Eisman, Anne J Søgaard, Nina Emaus

**Affiliations:** 1 Department of Community Medicine, Faculty of Health Sciences, University of Tromsø, Tromsø, 9037, Norway; 2 Garvan Institute of Medical Research, University of New South Wales, Sydney, Australia; 3 Department of Health and Care Sciences, University of Tromsø, 9037, Tromsø, Norway; 4 St. Vincent’s Hospital, Sydney, Australia; 5 Norwegian Institute of Public Health, Oslo, Norway

**Keywords:** Morphometry, Vertebral fractures, Risk factors, Population based study, Population attributable risk (par)

## Abstract

**Background:**

Vertebral fractures, the most common type of osteoporotic fractures, are associated with increased risk of subsequent fracture, morbidity, and mortality. The aim of this study was to examine the contribution of important risk factors to the variability in vertebral fracture risk.

**Methods:**

Vertebral fracture was ascertained by VFA method (DXA, GE Lunar Prodigy) in 2887 men and women, aged between 38 and 87 years, in the population-based Tromsø Study 2007/2008. Bone mineral density (BMD; g/cm^2^) at the hip was measured by DXA. Lifestyle information was collected by questionnaires. Multivariable logistic regression model, with anthropometric and lifestyle factors included, was used to assess the association between each or combined risk factors and vertebral fracture risk. Population attributable risk was estimated for combined risk factors in the final multivariable model.

**Results:**

In both sexes, age (odds ratio [OR] per 5 year increase: 1.32; 95% CI 1.19-1.45 in women and 1.21; 95% CI 1.10-1.33 in men) and BMD (OR per SD decrease: 1.60; 95% CI 1.34-1.90 in women and1.40; 95% CI 1.18-1.67 in men) were independent risk factors for vertebral fracture. At BMD levels higher than 0.85 g/cm^2^, men had a greater risk of fracture than women (OR 1.52; 95% CI 1.14-2.04), after adjusting for age. In women and men, respectively, approximately 46% and 33% of vertebral fracture risk was attributable to advancing age (more than 70 years) and low BMD (less than 0.85 g/cm^2^), with the latter having a greater effect than the former.

**Conclusions:**

These data confirm that age and BMD are major risk factors for vertebral fracture risk. However, in both sexes the two factors accounted for less than half of fracture risk. The identification of individuals with vertebral fracture is still a challenge.

## Background

Fragility fracture is highly prevalent in the general population, and is increasingly becoming a public health concern [1,2]. From the age of 60, the lifetime risk of any fracture in Norway is 63% in women and 34% in men [3]. Although hip fracture is the most serious consequence of osteoporosis [4], other osteoporotic fractures are also associated with important health issues as pain [5], loss of function [6], and increased risk of new fractures [7]. Some studies have suggested that clinical vertebral fractures are associated with increased risk of subsequent vertebral fractures [8-10], non-vertebral fractures [11-14], and mortality [15]. Vertebral fracture is often underestimated due largely to the problem of under-diagnosis. Indeed, only one in three vertebral fractures is clinically diagnosed [5,16], and the majority is either undetected or incidentally detected through radiographs. Recent development in dual x-ray absorptiometry (DXA) technology has allowed a population based assessment of vertebral fracture to be carried out by common DXA densitometers [17]. The vertebral fracture assessment (VFA) method has been used in many population settings, and its sensitivity and specificity are comparable to spinal radiographs in the ability to diagnose grade 2 and 3 (moderate and severe) vertebral fracture [13,17]. These developments provide opportunities for studies of vertebral fracture risk.

Norway is among the countries in the world with the highest incidence of fragility fractures, including hip [18] and forearm [19]. According to the FRAX risk calculator, age, sex, body mass index, previous fracture, parental hip fracture history, alcohol, tobacco, arthritis, and use of glucocorticoids constitute important risk factors for osteoporotic fractures [20]. Studies on vertebral fractures indicate that among these, age, bone mineral density (BMD), and previous fractures are the most significant risk factors [21,22]. Data recently reported from the Tromsø Study in Norway indicate that the prevalence of vertebral fractures do not differ between women and men [23]. The aim of the present study was therefore to address the important risk factors for vertebral fractures in a Norwegian population, and to estimate the percentage of fracture cases that can be explained by these risk factors.

## Methods

### Study population

The present study is part of the on-going Tromsø Study (http://www.tromsostudy.com). Details of study design have been described elsewhere [24]. Briefly, the study is a longitudinal, population-based investigation in Tromsø (Norway), a city of nearly 70000 inhabitants. The study comprises six repeated surveys from its start in 1974 (Tromsø I) through 2007/08 (Tromsø VI) [24]. Only men were invited to the first survey, but from Tromsø II 1979/80 both women and men have been included. The participation rate have ranged from 65% to 77% [24]. Each survey has been conducted in two phases, with the most basic examination in phase 1 (height, weight, blood pressure, blood samples, and questionnaires) and more extensive examinations for a random sub-sample of the cohort in phase 2, depending on available financial resources. The Tromsø Study, including this study, has been approved by the Norwegian Data Inspectorate and the Regional Committees for Medical and Health Research Ethics (Rec North). All participants signed a written informed consent.

The present study is based on data from Tromsø VI, details described elsewhere [23]. In short, 9625 men and 10137 women were invited, and 6054 men (62.9%) and 6930 women (68.4%) attended phase 1 of the survey. Among those, a total of 11484 subjects were invited for phase 2, and 3141 men (61.5%) and 4166 women (65.3%) attended. Persons with valid BMD measuremenst from Tromsø V in 2001/2002 were invited for BMD measurements of the hip, i.e. a dual femur scan, and altogether 3854 persons attended. Among these, a VFA, also called lateral vertebral assessment (LVA), was performed in a randomly selected group of 2894 persons. Seven blurred VFA scans had to be excluded, leaving 2887 persons, 1206 men and 1681 women, with clearly measurable VFA scans and total hip measurements. Among the 2887 persons with VFA scans, we obtained the following numbers according to each vertebral level: T4 = 2350, T5 = 2743, T6 = 2845, T7 = 2863, T8 = 2875, T9 = 2878, T10 = 2885, T11-L3 = 2887, L4 = 2848.

### Ascertainment of vertebral fracture

Vertebral fracture was ascertained by the VFA of the GE Lunar Prodigy, Lunar Corporation, Madison, WI, USA, version 12.20. Vertebral morphometry is a quantitative method developed for identification of osteoporotic vertebral fractures based on the measurement of vertebral heights, identifying the anterior, middle, and posterior heights of each vertebra. Depending on their relative relations according to a given reference, the software identifies three types of fractures: wedge, biconcave, and compression, according to three degrees of severity, ranging from mild through moderate to severe [25]. Although some authors suggest that spinal radiograph is the gold standard for the diagnosis of vertebral fractures [13,26], the morphometric method is recognized as being easy, precise and using low radiation exposure [25,27,28], with high precision in measuring moderate and severe deformities [13]. In our dataset, only 1% of the deformities were identified as being mild, the majority were either moderate or severe [23]. All our scans were taken according to the standard set by GE Lunar Prodigy, and specially trained technicians did the scanning according to the standardized protocol. One of the technicians (the first author) performed the quality assessment of the total material afterwards. For precision analysis of the VFA, a random sample of 50 participants was re-analyzed by the same technician. The mean intra-class correlation coefficient was 0.84 for average height of the vertebrae [23].

### Bone mineral density

BMD expressed as g/cm^2^ was measured at the total hip and femoral neck by DXA, using the same densitometer as for the VFA (GE Lunar Prodigy). Daily phantom measurements were performed throughout the survey. Three technicians did the scanning according to a standardized protocol, and one of them performed the quality assessment of the total material afterwards. The short term *in vivo* precision error was 1.2 and 1.7% for total hip and femoral neck measurements, respectively [29]. For the main analyses of this study, we have included BMD measurement of the total hip, where 2738 valid measurements were available (either left or right hip). Based on the Lunar reference, 102 persons (3.5%) had a T-score ≤ −2.5 (osteoporosis) and 789 persons (27%) had a T score between −2.5 and −1.0 (osteopenia).

### Questionnaire

Data on lifestyle variables were collected through questionnaires in both phases of the study. The questionnaires obtained information, among other, on smoking habits, physical activity, self-perceived health, and education. Smoking status was classified into three categories, namely: present, former, and never. These were further grouped into two categories: former and never smokers were categorized as “not smoking” and smokers as “smoking”. The question on leisure time physical activity level during the last year had four alternatives from sedentary (mostly tranquil activities), moderate (lightly active at least four hours a week), active (vigorously active at least four hours a week) and highly active (exercising regularly several times a week). The sedentary and moderate active were categorized into one “low active group”, and the active and highly active into one “high active” group. Five levels of self-perceived health (very good, good, neither good nor bad, bad, and very bad) were categorized into two, good (very good and good) and poor. Five levels of education were categorized into three levels: 1) primary school only (i.e. seven years), 2) up to four years more than primary school, and 3) more than four years after primary school.

### Data analysis

Each individual was classified as having a vertebral fracture if there was a presence of at least one fracture as described in the “Ascertainment of vertebral fracture” section. The association between sex and vertebral fracture risk was assessed by Chi-square statistics. Univariate analyses (Chi-square statistics or Independent sample T-tests) were used to examine the association between baseline characteristics and vertebral fracture risk in women and men. Logistic regression was used to assess the association between each risk factors and vertebral fracture risk in women and men separately, adjusting for age. Odds ratio (OR) of fracture (and 95% confidence interval (CI)) was estimated per standard deviation (SD) of continuous risk factors (e.g., BMD, body mass index (BMI)). Multivariable logistic regression analysis was used to assess the association between each significant or combined risk factors and vertebral fracture risk. Testing for interaction was done by including the product of sex and BMD and sex and age in the model. The final and most optimal model was found using backward selection procedures. In order to assess the impact of risk factors on vertebral fracture, we estimated the population attributable risk fraction (PAR) for combined risk factors in the “final” multivariable model, for women and men separately. In this study context, PAR represents the proportion of vertebral fractures that can be attributed to a risk factor, if there is a causal relationship between the risk factor and fracture [30]. However, because vertebral fracture is associated with multiple risk factors, stratified models were used to estimate the fracture risk in four categories of exposure and the heuristic approach described by Hanley, J.A. [31] was used to calculate the PAR. The formula used to calculate the attributable risk (AR) (actually called attributable fraction) was the following:$AR=\frac{\left(RR_{i}-1\right)\times Pct_{i}}{1+\sum_{i}^{n}\left(RR_{i}-1\right)\times Pct_{i}}$(where (n) is the total number of the categories, and (i) takes value of each category risk (RR) and percentage (Pct)). All statistical analyses were performed with the SPSS statistical package (versions 19).

## Results

The study cohort included 2887 individuals (1206 men and 1681 women) aged between 38 and 87 years, with the majority being post-menopausal (women) or aged 50+ years.

Overall, approximately 64% of men and 61% of women reported good health status, with a majority (more than 78%) in the sedentary or moderately physical activity group.

Vertebral fracture was found in 166 (13.8%) men and 199 (11.8%) women (p = 0.07). Baseline characteristics of participants stratified by fracture status and sex are shown in Table 1. Men and women with a vertebral fracture were on average older than those without and had lower total hip BMD. Among women, those with a fracture also had lower levels of education and poor overall self-reported health status compared to those without a fracture in addition to lower weight and stature. The results did not change when we included only participants above 50 years of age in analyses.

**Table 1:** Descriptive statistics by gender and morphometric vertebral fracture, the Tromsø Study 2007-08

**Gender and factor**	**No Fracture**	**Vertebral fracture**	**P-value**
**Women (N)**	**1482**	**199**	
Age (years)	64.7 (9.3)	70.5 (8.6)	<0.0001
Weight (kg)	71.1 (12.5)	68.4 (12.7)	0.005
Height (cm)	162.5 (6.3)	160.4 (7.1)	<0.0001
BMI (kg/m^2^)	26.9 (4.6)	26.6 (4.5)	0.297
Total hip BMD (g/m^2^)	0.91 (0.13)	0.83 (0.11)	<0.001
**Education**			0.009
Primary school (n; %)	627 (43.0)	106 (54.4)	
O-level	375 (25.7)	44 (22.6)	
More than O-level	456 (31.3)	45 (23.1)	
**Physical activity**			0.796
High active (n; %)	144 (11.2)	18 (10.5)	
Low active	1143 (88.8)	153 (89.5)	
**Smoking status**			0.750
Daily smokers (n; %)	265 (18)	37 (19)	
Non smoking	1189 (82)	156 (81)	
**Health status**			0.029
Good (n; %)	905 (61.9)	106 (53.8)	
Poor	557 (38.1)	91 (46.2)	
**Men (N)**	**1040**	**166**	
Age (years)	64.8 (9.3)	69.0 (9.2)	<0.0001
Weight (kg)	84.5 (12.3)	82.8 (11.5)	0.078
Height (cm)	175.5 (6.5)	174.4 (6.7)	0.062
BMI (kg/m^2^)	27.4 (3.5)	27.2 (3.4)	0.457
Total hip BMD (g/m^2^)	1.03 (0.14)	0.98 (0.15)	<0.001
**Education**			0.147
Primary school (n; %)	324 (31.9)	54 (33.5)	
O-level	287 (28.2)	55 (34.2)	
More than O-level	405 (39.9)	52 (32.3)	
**Physical activity**			0.577
High active (n; %)	213 (22.6)	31 (20.5)	
Low active	731 (77.4)	120 (79.5)	
**Smoking status**			0.988
Daily smokers (n; %)	159 (15.5)	25 (15.5)	
Non smoking	868 (84.5)	136 (84.5)	
**Health status**			0.574
Good (n; %)	662 (64.1)	102 (61.8)	
Poor	371 (35.9)	63 (38.2)	

*P-values refer to univariate analyses.

**Figure 1:**
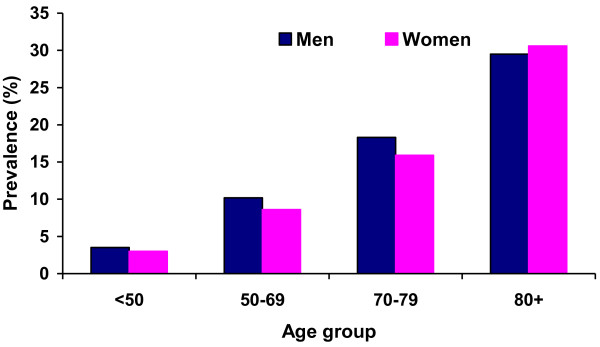
**The association between age and the prevalence of morphometric vertebral fracture in women and men, Tromsø Study 2007–08.** Age was significantly associated with the prevalence of vertebral fracture in women and men (p < 0.001).

In either sex, age was significantly associated with the prevalence of vertebral fracture (p < 0.001) (Figure 1). When data were combined and analyzed in a bivariate logistic regression model (Table 2), the risk of vertebral fracture increased with advancing age, OR 1.43; 95% CI: 1.31 to 1.56 in women and OR 1.28; 95% CI: 1.17 to 1.40 in men, per 5-year increase. Decreasing total hip BMD, increased body weight and higher stature were each associated with reduced vertebral fracture risk, but after adjusting for age, only BMD was associated with vertebral fracture risk in men (OR 1.40; 95% CI: 1.18 to 1.67 per −0.14 g/cm^2^ decrease), and in women only BMD (OR 1.60; 95% CI: 1.34 to 1.90 per −0.13 g/cm^2^ decrease) and weight (OR 0.93; 95% CI: 0.88 to 0.99 per 5 kg increase) (Table 2).

**Table 2:** The association between risk factors and morphometric vertebral fracture in bivariate analysis (logistic regression)

**Risk factor**	**Unit of comparison**	**OR and 95%CI**	**OR and 95%CI**
		**Women**	**Men**
Age	+5 years	1.43 (1.31 – 1.56)	1.28 (1.17 – 1.40)
Total hip BMD	−0.13/-0.14 g/cm^2*^	1.60 (1.34 – 1.90)	1.40 (1.18 – 1.67)
Weight	+5 kg	0.93 (0.88 – 0.99)	0.99 (0.92 – 1.06)
Height	+5 cm	0.89 (0.79 – 1.01)	0.97 (0.85 – 1.11)
BMI	+ 4 kg/m^2^	0.88 (0.74 – 1.04)	0.99 (0.78 – 1.27)
Health status	Good vs Poor	0.87 (0.64 – 1.19)	0.98 (0.70 – 1.38)
Physical activity	High vs Low	1.11 (0.65 – 1.89)	0.92 (0.60 – 1.41)
Smoking status	Yes vs No	0.77 (0.52 – 1.14)	0.88 (0.55 – 1.40)

**Note**: The effect of BMD, weight, height, etc. was each adjusted for age *-1 SD which was −0.13 g/cm^2^ in women and −0.14 g/cm^2^ in men.

**Figure 2:**
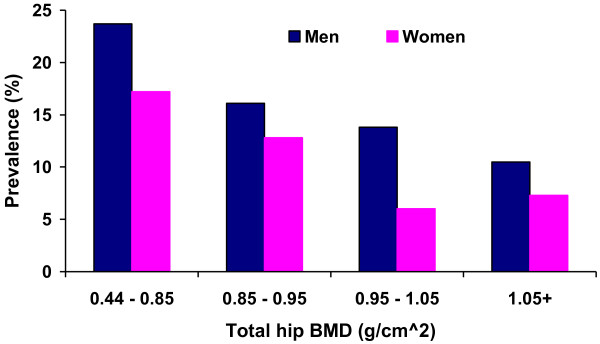
The association between BMD and the prevalence of morphometric vertebral fracture in women and men, Tromsø Study 2007–08.

Because sex was not associated with the prevalence of vertebral fractures in any age group (p > 0.12) (Figure 1), we wanted to explore the association between sex and vertebral fracture risk. BMD was inversely related to the prevalence of vertebral fracture in both sexes (p < 0.001) (Figure 2). After adjusting for age and BMD, the odds of vertebral fracture in men was 1.87-fold (95% CI 1.4 to 2.5) higher than in women. Testing for interaction between the variables showed that there was no interaction between sex and age (p = 0.08), but a significant interaction between sex and BMD (p = 0.005). According to the WHO definition [32], 1.5% of all men and 6% of all women had osteoporosis (T-score ≤ −2.5 at the total hip). Categorizing BMD into T-scores, men had greater risk of vertebral fracture than women in the normal and normal + groups (T scores > −1.5) (p < 0.012) and in the osteoporosis group (p = 0.011), but the numbers were low. We therefore categorized the BMD levels into quartiles, kept the lowest quartile of less than 0.85 g/cm^2^ as one group, and the rest as another. With this stratification, at BMD levels higher than 0.85 g/cm^2^, men had a greater risk of fracture than women (OR 1.52; 95% CI 1.14-2.04), after adjusting for age.

A multivariable logistic regression model was conducted to search for independent risk factors for each sex separately, and the results are shown in Table 3. For both women and men the “final” and most optimal model for predicting fracture risk included age and total hip BMD. For a given age group, the risk of fracture increased as BMD decreased. Conversely, for a given BMD category, the prevalence of fracture increased with advancing age. Attributable risk analysis (Table 4) indicated that in women 45.9% and in men 33.4% of all vertebral fractures cases were attributable to advancing age (i.e. more than 70 years) and lower total hip BMD (i.e. less than 0.85 g/cm^2^).

**Table 3:** Risk factors for morphometric vertebral fracture in multivariable analysis (This analysis was based on 2738 subjects, not 2887)

**Risk factor**	**Unit of comparison**	**OR and 95**% **CI**	**OR and 95**% **CI**
		**Women**	**Men**
Age	+5 years	1.32 (1.19 – 1.45)	1.21 (1.10 – 1.33)
Total hip BMD	−0.13/-0.14 g/cm^2*^	1.60 (1.34 – 1.90)	1.40 (1.18 – 1.67)

**Note**: All OR: p < 0.001 *-1 SD which was −0.13 g/cm^2^ in women and −0.14 g/cm^2^ in men.

**Table 4:** Determination of attributable risk (heuristic methods)

**Age**	**BMD**	**N**	**Percent**	**RR**	**PAR**
**Women**					
<70	0.85+	781	49.7	1.0	
	<0.85	296	18.8	1.72	7.4
70+	0.85+	227	14.4	2.01	7.9
	<0.85	267	17.0	4.33	30.6
All	All	1571	100		**45.9**
**Men**					
<70	0.85+	692	59.3	1.0	
	<0.85	49	4.2	1.79	2.2
70+	0.85+	357	30.6	2.00	20.4
	<0.85	69	5.9	3.75	10.8
All	All	1167	100		**33.4**

**Remark**: Approximately 46% of all vertebral fracture cases in women were attributable to old age and low BMD, with the latter - low BMD - (31%) having more pronounced effect than the former. In men, 33% of the fracture cases were attributable to old age and low BMD, with the former - old age - (20%) having more effect than the latter.

## Discussion

In line with other studies [21,22,33], we found that advancing age and lower BMD at the total hip were independent determinants of vertebral fracture risk, whereas all other background- and lifestyle variables did not contribute statistically in the final model. The estimated population attributable risk fraction for these two factors combined was 45.9% in women and 33.4% in men.

At the highest BMD levels, the risk of vertebral fracture at given age was greater in men than in women. Few population-based studies have included vertebral fracture assessment in both women and men. It is therefore difficult to compare the present sex-related vertebral fracture risk. There has been some controversy in the literature about whether there is a higher risk of vertebral fractures in women than in men. Our finding of non-significant difference in vertebral fracture risk between men and women (before adjustment of age and BMD) is not consistent with a previous review which suggested that the incidence of vertebral fractures in men is about one third to one half of that in women [34], but of course, our estimates are based on prevalent vertebral fractures. However, our results are in line with findings from the Dubbo Osteoporosis Epidemiological Study [35] where the prevalence of vertebral deformities was higher in men than in women. In that study the higher prevalence in men was observed regardless of diagnostic criteria, suggesting, as in our study, that vertebral fractures may be overlooked in men.

Several other studies report, similar to the present study, that low BMD measured at the femoral sites are associated with prevalent radiographic vertebral fractures [21,22,28]. We have not seen other studies comparing sex difference at normal and normal + levels (not osteoporosis nor osteopenia). The higher prevalence of vertebral deformities at these BMD levels in men indicate, as also suggested by others [16], that these deformities could be of other origin than osteoporosis, possibly mechanic or due to childhood diseases [34] and should be explored in follow up studies. Studies defining the proportion of fractures attributable to trauma in childhood and young adulthood are lacking [34]. As Seeman et al. claims, if these youth fractures do make a contribution to the numbers of elderly men with fractures, this will exaggerate the prevalence of vertebral fractures and by that mask a sex difference in the prevalence of osteoporotic vertebral fractures [34], something that should be examined in follow-up studies. The finding from our study indicating that at a given age and given BMD level the OR for having a vertebral fracture is higher in men than in women, somehow corresponds to how the risk of hip fractures seems to be similar in men and women for any given BMD [16,32,36,37]. Although an unknown proportion of the vertebral fractures in men may be of mechanic origin, our study highlights the importance of osteoporosis and vertebral fracture risk in men, and that vertebral fractures should definitely not be considered a women health problem only.

The present findings should be viewed within the context of strengths and potential limitations. The Tromsø Study is a population-based, longitudinal study with a high participation rate. The present study is a cross-sectional survey within the framework of the Tromsø Study, where vertebral morphometry was done for the first time. Because of its cross-sectional design, causal inference cannot be drawn from the findings and the results will need confirmation within a longitudinal design. For logistic reasons, we did not perform quality control using x-ray technology. It is, however, reported that DXA scans are more precise in measuring moderate and severe than mild deformities [13], and 99% of the identified deformities in our data set were either moderate or severe. The intra-class correlation coefficient for determination of average height of the vertebra was good. Ideally, we should have compared determination of fracture severity in the sample. This was, however, difficult to attain, since determination is done electronically by the software, based on identified vertebral heights. Despite of random selection in the Tromsø Study, the morphometry group was younger with a slightly lower proportion of women compared to the group not selected to VFA. However, when we compare the morphometry group with the phase 2 participants of the Tromsø VI survey, whom to our best knowledge should be a representative sample [24], the morphometry sample of women and men was slightly older (3 years) with lower height (2 cm), but did not differ significantly in any other way. Despite high rates of hip- and forearm fractures in Norway, the prevalence of vertebral fractures in this population was comparable to reports from others [38-40]. Although we should be careful drawing firm conclusions from our prevalence estimates, we still feel comfortable to compare between women and men, especially in age stratified analyses. It is a major limitation to our study that we lack information of important risk factors included in FRAX [20], especially the history of vertebral fractures [21,33]. Although BMI [41] and smoking [42] are considered to be independent predictors of fracture risk, they did not contribute to the final model. The predictive value of physical activity, self-perceived health, and education to fracture risk is uncertain [43] and did not affect the results although the self-reported nature and our dichotomization of the variables may have precluded possible associations. However, this study confirms that age and BMD are important predictors of vertebral fractures in women and men [21,22].

## Conclusion

Advancing age and declining BMD are independent determinants of vertebral fracture risk in women and men, but accounting for less than half of the total risk. The predictive value of prevalent vertebral fractures on subsequent vertebral fractures and other types of fractures should be explored in longitudinal studies.

## Competing interests

The authors declare that they have no competing interests.

## Authors’ contributions

Contributions of the authors to the manuscript included *Study concept and design*: NE, SW, AJS; *Acquisition of data:* NE, SW, NDN, LAA; *Analyses and interpretation of data*: SW, NE, LAA, AJS, JC, JAE; *Statistical analyses*: SW, NE, LAA, TN; *Critical revision of the manuscript*: SW, LAA, AJS, JC, NDN, TN, JAE, NE. All authors read and approved the final manuscript.

## Pre-publication history

The pre-publication history for this paper can be accessed here:

http://www.biomedcentral.com/1471-2474/13/163/prepub
